# Activities of Daily Living Associated with Acquisition of Melioidosis in Northeast Thailand: A Matched Case-Control Study

**DOI:** 10.1371/journal.pntd.0002072

**Published:** 2013-02-21

**Authors:** Direk Limmathurotsakul, Manas Kanoksil, Vanaporn Wuthiekanun, Rungrueng Kitphati, Bianca deStavola, Nicholas P. J. Day, Sharon J. Peacock

**Affiliations:** 1 Department of Tropical Hygiene, Faculty of Tropical Medicine, Mahidol University, Bangkok, Thailand; 2 Mahidol-Oxford Tropical Medicine Research Unit, Faculty of Tropical Medicine, Mahidol University, Bangkok, Thailand; 3 Department of Pediatrics, Sappasithiprasong Hospital, Ubon Ratchathani, Thailand; 4 Bureau of Emerging Infectious Diseases, Department of Disease Control, Ministry of Public Health, Nonthaburi, Thailand; 5 Department of Medical Statistics, Faculty of Epidemiology and Population Health, London School of Hygiene and Tropical Medicine, London, United Kingdom; 6 Centre for Clinical Vaccinology and Tropical Medicine, Nuffield Department of Clinical Medicine, University of Oxford, Churchill Hospital, Oxford, United Kingdom; 7 Department of Microbiology and Immunology, Faculty of Tropical Medicine, Mahidol University, Bangkok, Thailand; 8 Department of Medicine, Cambridge University, Addenbrooke's Hospital, Cambridge, United Kingdom; University of Tennessee, United States of America

## Abstract

**Background:**

Melioidosis is a serious infectious disease caused by the Category B select agent and environmental saprophyte, *Burkholderia pseudomallei*. Most cases of naturally acquired infection are assumed to result from skin inoculation after exposure to soil or water. The aim of this study was to provide evidence for inoculation, inhalation and ingestion as routes of infection, and develop preventive guidelines based on this evidence.

**Methods/Principal Findings:**

A prospective hospital-based 1∶2 matched case-control study was conducted in Northeast Thailand. Cases were patients with culture-confirmed melioidosis, and controls were patients admitted with non-infectious conditions during the same period, matched for gender, age, and diabetes mellitus. Activities of daily living were recorded for the 30-day period before onset of symptoms, and home visits were performed to obtain drinking water and culture this for *B. pseudomallei*. Multivariable conditional logistic regression analysis based on 286 cases and 512 controls showed that activities associated with a risk of melioidosis included working in a rice field (conditional odds ratio [cOR] = 2.1; 95% confidence interval [CI] 1.4–3.3), other activities associated with exposure to soil or water (cOR = 1.4; 95%CI 0.8–2.6), an open wound (cOR = 2.0; 95%CI 1.2–3.3), eating food contaminated with soil or dust (cOR = 1.5; 95%CI 1.0–2.2), drinking untreated water (cOR = 1.7; 95%CI 1.1–2.6), outdoor exposure to rain (cOR = 2.1; 95%CI 1.4–3.2), water inhalation (cOR = 2.4; 95%CI 1.5–3.9), current smoking (cOR = 1.5; 95%CI 1.0–2.3) and steroid intake (cOR = 3.1; 95%CI 1.4–6.9). *B. pseudomallei* was detected in water source(s) consumed by 7% of cases and 3% of controls (cOR = 2.2; 95%CI 0.8–5.8).

**Conclusions/Significance:**

We used these findings to develop the first evidence-based guidelines for the prevention of melioidosis. These are suitable for people in melioidosis-endemic areas, travelers and military personnel. Public health campaigns based on our recommendations are under development in Thailand.

## Author Summary

Melioidosis is a serious infectious disease caused by the environmental saprophyte, *Burkholderia pseudomallei*. The infection is potentially preventable, but developing prevention guidelines is hampered by a lack of evidence on which to base them. The purpose of this study was to provide evidence for inoculation, inhalation and ingestion as routes of infection. To achieve this, we undertook a matched case-control study and performed home visits to obtain drinking water and culture this for *B. pseudomallei*. We found that activities associated with increased risk of developing melioidosis included working in a rice field, other activities associated with exposure to soil or water, an open wound, eating food contaminated with soil or dust, drinking untreated water, outdoor exposure to rain, water inhalation, current smoking and steroid intake. Presence of *B. pseudomallei* in drinking water source(s) doubled the odds of acquiring melioidosis. This is the first study to show that ingestion is an important route of human *B. pseudomallei* infection, and that exposure to rain is an independent risk factor for melioidosis. We used this finding to develop the first evidence-based guidelines for the prevention of melioidosis. These are suitable for people in melioidosis-endemic areas, travelers and military personnel.

## Introduction

*Burkholderia pseudomallei* is a Category B select agent and the cause of naturally acquired melioidosis in South and East Asia, Northern Australia, the Indian subcontinent and areas of South America [1]–[3]. Northeast Thailand is a hotspot for this infection, with an annual incidence of 21.0 per 100,000 population and a crude mortality rate of 40% [4]. This rate is comparable to that for deaths from tuberculosis in this region, where melioidosis is the third most common cause of death from infectious diseases [4]. Visitors to areas where melioidosis is endemic are also at risk of acquiring this infection. Melioidosis is readily misdiagnosed in returning travelers because of a lack of familiarity with the clinical and microbiological features, compounded by a highly variable incubation period that may extend to many decades [5], [6]. The largest transient population to have been affected in living memory was US combatants in the conflict with Vietnam, when the disease acquired the nickname ‘Vietnamese time bomb’ [7].

*B. pseudomallei* is present in soil and surface water in areas where melioidosis is endemic, and most cases are thought to result from bacterial inoculation [8]. This is based on the observations that people at high risk of melioidosis such as agricultural workers in Thailand and indigenous people in Australia are regularly exposed to soil and water without protective clothing and may suffer repeated minor injuries [9], [10]. The role of other routes of infection is uncertain. Inhalation may have been a route of infection for US combatants during the Vietnam conflict [11], and several studies from northern Australia have reported a shift towards a higher frequency of pneumonia and severe disease during the rainy season or following heavy monsoon rains and winds [12]–[14]. Recent evidence also suggests that ingestion might be an important route of *B. pseudomallei* infection. West et al. showed that gastric inoculation of *B. pseudomallei* led to melioidosis in an experimental mouse model [15]. Several clusters of melioidosis cases have been reported from Australia in which a strain of *B. pseudomallei* isolated from a common water source was a genetic match for the strain causing disease in the cluster [16], [17], although it is not clear whether these cases were infected through ingestion rather than inoculation.

Melioidosis is potentially preventable, but developing prevention guidelines is hampered by a lack of evidence on which to base them. Advice in Northern Australia is based on common sense and includes avoidance of direct contact with soil and standing water and washing after exposure [18]. There are no recommendations to prevent melioidosis via inhalation or ingestion. No advice is given in Asia or other places where melioidosis is endemic, and no advice is given to tourists despite the steady trickle of cases in returning travelers. Here, we describe a matched case-control study in which we identify activities associated with an increased risk of disease acquisition, define the importance of three routes of melioidosis infection, and describe the first evidence-based guidelines for the prevention of melioidosis.

## Methods

### Setting and study design

A prospective 1∶2 matched case-control study was performed at Sappasithiprasong Hospital between Jul 2010 and Dec 2011. This 1,100-bed hospital is situated in the provincial town of Ubon Ratchathani in northeast Thailand, 70 km west of Laos and 95 km north of Cambodia, and serves around 2 million people. Cases were initially identified through daily contact with the hospital diagnostic microbiology laboratory, and were defined as patients aged ≥18 years with culture-proven melioidosis (isolation of *B. pseudomallei* from any clinical sample and compatible clinical features). Controls were identified through the hospital computerized admission records, and were defined as patients admitted with non-infectious conditions during the same period (+/−2 weeks, and therefore season), matched for gender, age (+/−5 years), and presence or absence of diabetes mellitus. Patients admitted with infectious conditions were not eligible as controls, as the sensitivity of culture for the diagnosis of melioidosis is not perfect [19]. As a result, culture-negative melioidosis patients were not enrolled as controls. Matching was performed for known predisposing factors (diabetes, gender, age and time of presentation) to control for confounding. Target enrollment numbers were at least 250 cases and 500 controls, which would allow the detection of an approximate odds ratio of 2.0 with 90% power using a two-sided 1% test [19].

Each case and control was interviewed and information collected on specified activities of daily living during the 30 days preceding the onset of symptoms using a standardized study form. Relatives were interviewed if patients were not capable of answering questions. Patients with melioidosis are often severely unwell, and complete data capture via relatives was considered important to avoid the bias associated with exclusion of this group (Text S1). Trained study staff administered the questionnaire. The study was approved by the research ethics committees of Sappasithiprasong Hospital, and the Faculty of Tropical Medicine, Mahidol University. Written informed consent was obtained from all participants. Further details of the study design, definitions of cases and controls, assessment of exposure and statistical methods are provided in the supporting information.

### Sampling and culture of drinking water

A home visit was performed for case and control patients who resided within 100 km of the hospital, a distance limit imposed by the feasibility of travel, sample and data collection in the course of a single day. Five liters was collected from each source of drinking water, and tap water regardless of consumption. If the water was filtered or boiled by the householder before consumption, samples of these were collected for culture. Water samples were transported on the same day to our research laboratory at Sappasithiprasong Hospital and cultured for the presence of *B. pseudomallei*. In brief, for each 5 liter sample, 1 liter was passed through two 0.45 µm filters and 4 liters was passed through 2.5 g of sterile diatomaceous earth (Celite, World Minerals, USA) [20]. Filters were cultured on Ashdown agar to provide a quantitative bacterial count, and diatomaceous earth was cultured in selective broth (TBSS-C50) [21] to provide a sensitive, qualitative method. Broth was incubated at 40°C in air for 48 hours, after which 10 µl of the upper layer was streaked onto an Ashdown agar plate to achieve single colonies, incubated at 40°C in air and examined every 24 hours for 7 days. In the event that enrichment broth was positive but filters on Ashdown agar were negative, the quantitative count was defined as <1 CFU/L. Identification of bacterial colonies was performed as described previously [22].

### Statistical analysis

Univariable and multivariable conditional logistic regression analyses were performed. All variables that were either statistically significant in univariable analyses (with 0.25 significance level) or that were selected a priori based on current knowledge were included in multivariable analyses. The final multivariable model was developed using a purposeful selection method [23]. Data were analyzed using Stata12.0 (StataCorp, Texas, US). Conditional odds ratios are presented, and all p-values are two-tailed.

## Results

### Study subjects

**Figure 1:**
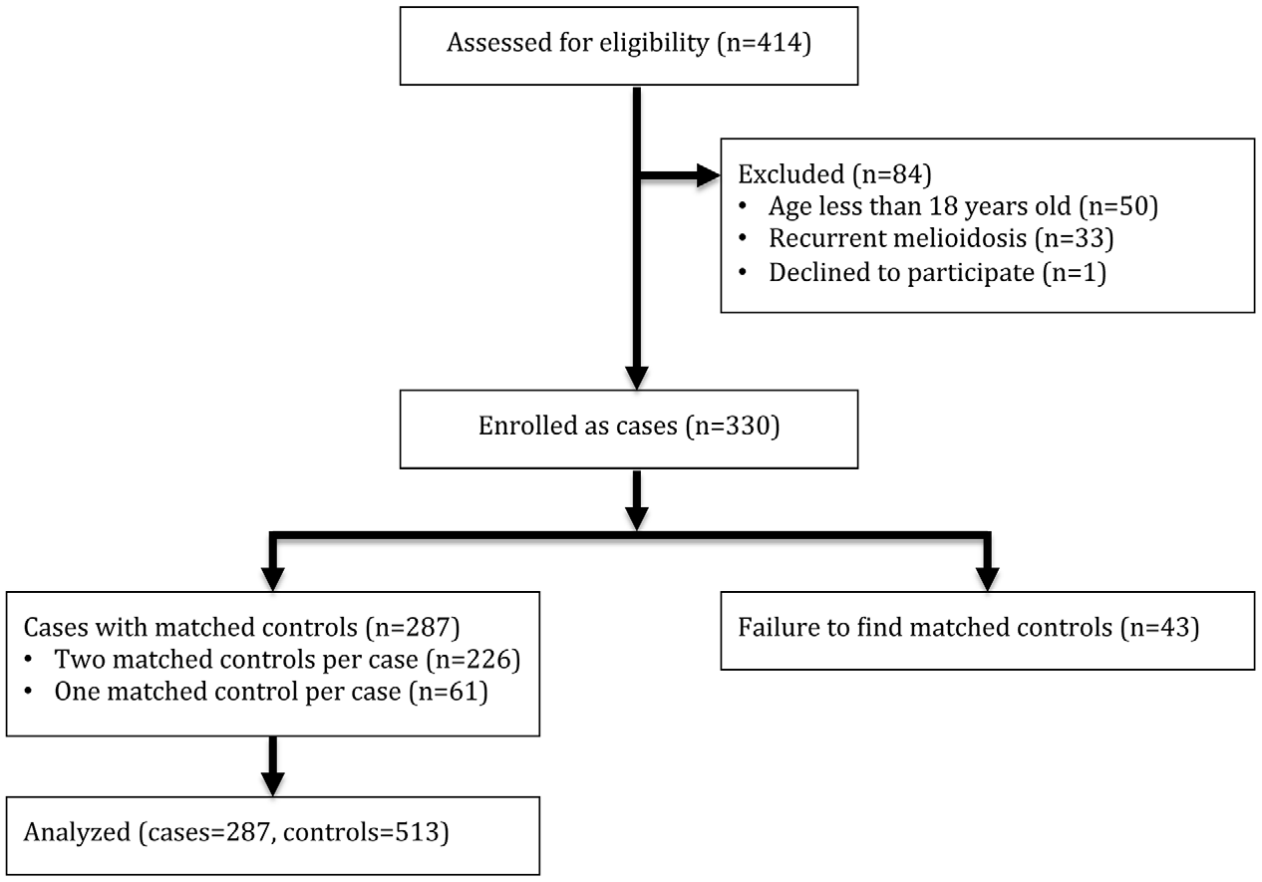
Study flow diagram.

A total of 414 patients presenting to Sappasithiprasong Hospital with culture confirmed melioidosis between July 2010 and December 2012 were assessed for eligibility (Figure 1). Of these, 84 patients were excluded because they were less than 18 years of age (n = 50), had recurrent melioidosis (n = 33), or declined to participate (n = 1).

Of the 330 cases enrolled into the study, two matched controls were identified for each of 226 cases (69%) and one matched control for 61 cases (18%). No matched control could be identified for the remaining 43 cases (13%) who were excluded from further analysis, giving a total of 287 cases and 513 controls. A history of activities of daily living prior to the onset of infective symptoms was obtained from relatives for a total of 92 cases (32%) and 26 controls (5%). A diagnosis of diabetes was more common in patients with melioidosis who were excluded because of failure to find a control, compared with those enrolled as cases (72% v.s. 42%, Table S1). The median age of cases was 54 years (interquartile range 46–64 years, range 18–88 years), 181 (63%) were male, 120 (42%) were diabetic, and 100 (35%) died within 28 days of the admission date.

The 513 controls were enrolled from various departments including surgery, orthopedics, general internal medicine and ophthalmology (Table S2). Common causes of illness were cancer (n = 58), bone fracture (n = 35), corneal ulcer (n = 26), cerebrovascular diseases (n = 20), cataract (n = 17), glaucoma (n = 17), calculus formation in the kidney or ureter (n = 16), and intervertebral disc disorder (n = 15).

### Activities of daily living

Working in a rice field in the month prior to the onset of infective symptoms was reported in 72% of cases and 48% of controls (Table S3), and almost tripled the odds of acquiring melioidosis (conditional odds ratio [cOR] 2.9, 95% confidence interval [CI] 2.1–4.0). The odds of having melioidosis increased by approximately 10% for each 10 working hours/week increase (cOR 1.1, 95%CI 1.0–1.1), and by approximately 20% for each 10 centimeter increase in depth that the legs were submerged in soil or water (cOR 1.2, 95%CI 1.0–1.3). Conversely, there was a decreased risk associated with wearing long trousers or rubber boots. There was no significant reduction in risk associated with wearing cloth gloves, and wearing rubber gloves was not reported. Washing after working in the rice field was associated with a decreased risk, but washing with water pooled in the rice field was associated with an increased risk of melioidosis (Table S3). Other activities leading to exposure to soil or water were also strongly associated with a risk of melioidosis (cOR 1.8, 95%CI 1.3–2.5 and cOR 2.3, 95%CI 1.7–3.3, respectively). People who walked barefoot everyday had nearly 2.5 times the odds of developing melioidosis compared to those who never walked barefoot (cOR 2.4, 95%CI 1.1–5.5). People who bathed in pond water had 11 times the odds of having melioidosis (cOR 11.1, 95%CI 1.3–92.5). Having an open wound was strongly associated with risk (cOR 2.4, 95%CI 1.4–4.1), and the risk increased if herbal remedies or an organic substance was applied directly onto an open wound (cOR 2.9, 95%CI 1.6–5.3).

Eating food contaminated with soil or dust was reported by 40% of cases and 22% of controls (cOR 2.4, 95%CI 1.7–3.3). Table S4 shows the water drinking habits of the 800 participants. Overall, 17% filtered and 13% boiled water before drinking. Following inspection of filtration machines, these were not considered to represent adequate treatment because of poor machine maintenance. Therefore, only bottled and boiled water was considered treated, while unboiled water from wells, boreholes, collected rainwater and tap water was considered untreated. Drinking untreated water was reported by 85% of cases and 72% of controls, and was associated with a doubling in the odds of acquiring melioidosis (cOR 2.3, 95%CI 1.5–3.3).

Outdoor exposure to a dust cloud or rain was associated with increased risk (cOR 1.6, 95%CI 1.2–2.2 and cOR 2.9, 95%CI 2.0–4.1, respectively). The use of a protective item (a mask or umbrella) was associated with a lower risk of infection although this did not reach significance (Table S4). Water inhalation of untreated water (accidental choking during drinking or swimming, associated with vigorous coughing) was reported by 23% of cases compared with 9% of controls (cOR 3.0, 95%CI 2.0–4.5). Being an active smoker was associated with increased risk (cOR 2.2, 95%CI 1.4–3.7), but being an ex-smoker was not (Table S4).

Consumption of any oral steroid medication was associated with a cOR of 3.2 (95%CI 1.6–6.3). Education beyond primary school was reported by 15% of cases and 26% of controls. A monthly income of greater than 5,000 baht per month was reported by 24% of cases and 37% of controls. Both factors were associated with halving the odds of acquiring melioidosis in the univariable model (Table S4).

### Multivariable conditional logistic regression

The final multivariable conditional logistic regression model included 286 cases and 512 controls (1 case and 1 control were excluded because of missing values). The findings of this analysis indicated that activities associated with an increased risk of melioidosis involved all three routes of acquisition. Working in a rice field, other activities leading to exposure to soil or water, eating contaminated food, drinking untreated water, outdoor exposure to rain, an open wound, water inhalation and taking steroids were independent risk factors in the final model (Table 1). There was borderline evidence that active smoking was associated with acquiring melioidosis (cOR 1.5, 95%CI 1.0–2.3, p = 0.069).

**Table 1:** Multivariable analysis of risk factors for melioidosis.

Activities	Conditional OR (95%CI)	P value
***Activities related to skin inoculation***		
No activities involving exposure to soil or water	1.0	0.003
Working in a rice field	2.1 (1.4–3.3)	
Other activities involving exposure to soil or water	1.4 (0.8–2.6)	
Open wound	2.0 (1.2–3.3)	0.005
***Activities related to ingestion***		
Eating food contaminated with soil or dust	1.5 (1.0–2.2)	0.045
Drinking untreated water	1.7 (1.1–2.6)	0.03
***Activities related to inhalation***		
Outdoor exposure to dust cloud	1.3 (0.9–1.8)	0.23
Outdoor exposure to rain	2.1 (1.4–3.2)	<0.001
History of water inhalation	2.4 (1.5–3.9)	<0.001
***Other risk factors***		
Current smoker	1.5 (1.0–2.3)	0.069
Taking oral steroids	3.1 (1.4–6.9)	0.006

Estimated odds ratios (OR) are conditional on the matching variables (gender, age, admission date (+/−2 weeks), and diagnosis of diabetes mellitus) and adjusted for the other risk factors included in the model.

### *B. pseudomallei* in drinking water

Home visits and sampling of drinking water was performed in 142/287 cases (49%) and 228/513 controls (44%) who resided within 100 kilometers of the hospital. *B. pseudomallei* was detected in 12% (10/84) of borehole water samples, 12% (32/273) of tap water samples, and 4% (1/27) of well water samples. *B. pseudomallei* was not detected in rain water (which is collected into a closed earthenware containers), or bottled water (0/160 and 0/32, respectively) (Table S5). The median quantitative count of *B. pseudomallei* in culture-positive samples was 1 CFU/L (interquartile range [IQR] <1 to 13; range <1 to 65 CFU/L). Two out of 53 samples of water that had been treated by a household member using filtration were culture positive for *B. pseudomallei*. Combining the results from the interview and microbiological data, we found that 7% (10/142) of cases and 3% (7/228) of controls drank water from sources that were demonstrated to contain *B. pseudomallei* (cOR 2.2, 95%CI 0.8–5.8).

### Guidelines for the prevention of melioidosis

On the basis of our findings, we propose that protection is required against all three routes of *B. pseudomallei* acquisition. We recommend that residents and visitors to melioidosis-endemic areas avoid direct contact with soil and water, outdoor exposure to heavy rain or dust clouds, do not consume untreated water, and wash food to be eaten raw using boiled or bottled water (Table 2). If direct contact with soil or water is necessary, we recommend that protective gear such as rubber gloves and boots or waders should be worn. We encourage cessation of smoking (particularly in those with underlying conditions such as diabetes that are known predisposing factors for melioidosis), and discourage the application of herbal remedies or organic substances to wounds.

**Table 2:** Recommendations for the prevention of melioidosis.

1. Avoid direct contact with soil or environmental water.
2. If contact with soil or environmental water is necessary, wear protective gear including rubber gloves, boots or waders, and wash with soap and clean water immediately after exposure.
3. In the event of an injury involving contamination with soil or environmental water, immediately clean the wound with soap and clean water.
4. Keep open wounds covered and avoid contact with soil or water until completely healed. Do not apply any herbal remedies or other substances to the wound. In the event that the wound comes into contact with soil or environmental water, clean the wound thoroughly with soap and clean water.
5. Always wear shoes. Do not walk bare foot.
6. Only drink bottled or boiled water. Do not drink any untreated water.
7. Do not eat food contaminated with soil or dust. If food is to be eaten without cooking, wash thoroughly using clean water. Use clean eating utensils, and wash these in clean water.
8. When outside, avoid heavy rain or dust clouds. If caught in a dust cloud, cover mouth and nose. Use an umbrella to protect yourself from the rain.
9. Do not smoke.
10. Be aware that you are at greater risk of melioidosis if you have certain conditions, including diabetes, chronic kidney disease, and diseases that require steroid therapy or medications that suppress the immune system.

## Discussion

This study has provided evidence to indicate that ingestion and inhalation, together with inoculation, are important routes for the development of melioidosis in Thailand. A range of activities were found to be independently associated with melioidosis, including presumed inoculation during unprotected occupational exposure to soil or environmental water, ingestion by eating contaminated food or drinking untreated water, and inhalation by outdoor exposure to rain. We also confirmed the presence of *B. pseudomallei* in water obtained from wells and boreholes and from piped water supplies, and recorded that a number of cases had consumed untreated water from these sources prior to presentation with melioidosis.

This is the first study to show that ingestion is an important route of human *B. pseudomallei* infection. Based on data obtained from The Provincial Waterworks Authority of Ubon Ratchathani province, only people living in the town of Ubon Ratchathani (3% of the provincial population) receive piped chlorinated water [24]. Tap water quality control does not include assessment for the presence of *B. pseudomallei*, which we found in 12% of tap water samples (32/273) and which a number of cases had consumed without adequate treatment. Unlike observations made in Hong Kong (14), none of the collected rainwater samples tested positive for *B. pseudomallei*. It is customary for the drinking rain water to be collected in large earthenware pots situated close to the house, the water in which can reach temperatures in excess of 40°C. This may explain the negative culture results in our setting, although it is also possible that the bacterial count was below the level of detection of our methodology. We recommend that all non-bottled water should be boiled prior to consumption. Although filtration is an alternative method of water purification, we observed that filters were poorly maintained and detected *B. pseudomallei* in some filtered water samples. In view of this, we do not recommend the use of filtration.

This is also the first evidence to indicate that exposure to rain is an independent risk factor for melioidosis. Exposure to dust clouds was a significant risk on univariable but not multivariable analysis. This is the first study to identify that smoking may be associated with acquiring melioidosis. Smoking could decrease the effectiveness of the local inflammatory response and increase the risk of infection by inhalation. Although microbiological confirmation of aerosolized *B. pseudomallei* has not been published, this could be due to poor sensitivity of the techniques used or a very low bacterial concentration. In experimental mice, inhalation of only 5 CFU can result in death within a few days [25].

Our study has several limitations. Relatives were asked about activities of daily living when cases or controls were not capable of providing this information, and it is possible that they were not aware of the full spectrum of activities undertaken. It is also possible that cases who were aware of their diagnosis of melioidosis might mention risk factors more readily than controls. This would only be the case if people were knowledgeable about melioidosis, but in a recent survey most Thai people (72%) had not heard of melioidosis, and the remainder had heard of the word but did not know what it meant (unpublished data). The education about melioidosis was given to all participants after the interview. There may be other factors associated with a risk of melioidosis that we failed to examine, and we cannot evaluate the relative risk of a matched variable. The study was not powered to identify risk factors with a relative risk less than 2.0. The criteria specified for matching were stringent and we were unable to find controls for some patients. A diagnosis of diabetes was more common in patients with melioidosis who were excluded because of failure to find a control, compared with those enrolled as cases (72% v.s. 42%). This is because the prevalence of diabetes in patients admitted to the hospital with non-infectious conditions (potential controls) was low, and finding matched controls for diabetic cases was more difficult than that for non-diabetic cases. However, diabetes is the strongest risk factor for melioidosis [8], and matching for diabetes is very important to control the possible confounding effect. We consider it likely that our findings are applicable to similar settings in neighboring Asia but may be less applicable to more distant geographical settings including Australia.

Current efforts are being directed toward increasing public awareness and implementing preventive measures for melioidosis in endemic areas, particularly Thailand. A vaccine that protects against *B. pseudomallei* infection is not available and there is no prospect of one being developed and ready for use in the near future [26]. There is, therefore, every reason to look for alterative solutions to prevent melioidosis, both in people living in regions of the world that are endemic for melioidosis, and visitors (e.g. travelers and military personnel) to these regions. The Ministry of Public Health in Thailand has included melioidosis on a priority list of emerging diseases in Thailand, and public health campaigns for melioidosis prevention based on the knowledge of this work are being developed. These actions will include the implementation of an education programme based on the recommendations provided here, the improvement of infrastructure relating to effective treatment of public water supplies and access to protective clothing, tractors and other machinery to reduce contact time of farmers with soil and environmental water. Further studies are required to model the cost-benefit of guidelines for the prevention of melioidosis, together with their acceptability, up-take and impact on rates of melioidosis.

## Supporting Information

Table S1 Characteristics of patients with culture confirmed melioidosis.
(DOC)

Table S2 Characteristics of matched controls.
(DOC)

Table S3 Activities associated with melioidosis acquisition by inoculation in the 30 days before onset of symptoms.
(DOC)

Table S4 Activities associated with melioidosis acquisition by ingestion and inhalation in the 30 days before onset of symptoms, and other risk factors.
(DOC)

Table S5 Sources of water consumed and presence of *B. pseudomallei* in drinking water.
(DOC)

Text S1 Supplementary methods.
(DOC)

Text S2 STROBE checklist.
(DOCX)
