# Supplementary material for: Activities of Daily Living Associated with Acquisition of Melioidosis in Northeast Thailand: A Matched Case-Control Study
Source: PLoS Negl Trop Dis. 2013 Feb 21;7(2):e2072. doi: 10.1371/journal.pntd.0002072 (PMC3578767; doi:10.1371/journal.pntd.0002072)
Supplement: Table S1 — Characteristics of patients with culture confirmed melioidosis. (DOC) [file pntd.0002072.s001.doc]

**Table S1.** Characteristics of patients with culture confirmed melioidosis

| **Factors** | **Cases enrolled into the study**  (n=287) | **Cases excluded due to lack of matched controls**  (n=43) | **P value** |
| --- | --- | --- | --- |
| Gender – % (no.) |  |  |  |
| Male | 63% (181) | 70% (30) | 0.50 |
| Female | 37% (106) | 30% (13) |  |
| Age – yr ^^ | 54 (IQR 46 to 64) | 51 (IQR 40 to 56) | 0.04 |
| Diabetes – % (no.) |  |  |  |
| Yes | 42% (120) | 72% (31) | < 0.001 |
| No | 58% (167) | 28% (12) |  |
| Mortality outcome – % (no.) |  |  |  |
| Died within 28 days after admission |  |  |  |
| Yes | 35% (100) | 44% (19) | 0.24 |
| No | 65% (187) | 56% (24) |  |
| Died within 2 days after admission |  |  |  |
| Yes | 15% (42) | 19% (8) | 0.49 |
| No | 85% (245) | 81% (35) |  |

^^Continuous variables are presented with the interquartile range (IQR).
