# Supplementary material for: Activities of Daily Living Associated with Acquisition of Melioidosis in Northeast Thailand: A Matched Case-Control Study
Source: PLoS Negl Trop Dis. 2013 Feb 21;7(2):e2072. doi: 10.1371/journal.pntd.0002072 (PMC3578767; doi:10.1371/journal.pntd.0002072)
Supplement: Table S2 — Characteristics of matched controls. (DOC) [file pntd.0002072.s002.doc]

**Table S2.** Characteristics of matched controls

| **Factors** | **Controls** (n=513) |
| --- | --- |
| Gender – % (no.) |  |
| Male | 63% (325) |
| Female | 37% (188) |
| Age – yr ^^ | 55 (IQR 45 to 63) |
| Diabetes – % (no.) |  |
| Yes | 36% (187) |
| No | 64% (326) |
| In-hospital mortality – % (no.) |  |
| Yes | 0% (1) |
| No | 100% (512) |
| Causes of illnesses – % (no.) |  |
| Diseases of the eye and adnexa (ICD10: H00-H59) | 14% (73) |
| Corneal ulcer (n=26) |  |
| Cataract (n=17) |  |
| Glaucoma (n=17) |  |
| Other diseases of the eye and adnexa (n=13) |  |
| Diseases of the musculoskeletal system and connective tissue (ICD10: M00-M99) | 14% (73) |
| Intervertebral disc disorders (n=15) |  |
| Fibroblastic disorders (n=14) |  |
| Spondylopathies (n=14) |  |
| Deforming dorsopathies (n=5) |  |
| Other diseases of the musculoskeletal system and connective tissue (n=25) |  |
| Neoplasms (ICD10: C00-C99) | 11% (58) |
| Malignant neoplasm of breast (n=11) |  |
| Malignant neoplasm of liver (n=8) |  |
| Malignant neoplasm of unidentified sites (n=8) |  |
| Other neoplasms (n=31) |  |
| Injury and certain other consequences of external causes (ICD10: S00-S99) | 11% (56) |
| Bone fracture (n=35) |  |
| Injury of eye and orbit (n=9) |  |
| Other injury and certain other consequences of external causes (n=12) |  |
| Diseases of the genitourinary system (ICD10: N00-N99) | 10% (53) |
| Calculus of kidney and ureter (n=16) |  |
| Chronic kidney disease (n=11) |  |
| Obstructive and reflux uropathy (n=6) |  |
| Other diseases of the genitourinary system (n=20) |  |
| Diseases of the digestive system (ICD10: K00-K99) | 9% (46) |
| Acute appendicitis (n=7) |  |
| Peptic ulcer (n=7) |  |
| Cholelithiasis (n=6) |  |
| Inguinal hernia (n=6) |  |
| Other diseases of the digestive system (n=20) |  |
| Diseases of the circulatory system (ICD10: I00-I99) | 7% (36) |
| Cerebrovascular diseases (n=20) |  |
| Other diseases of the circulatory system (n=16) |  |
| Other diseases | 36% (187) |

^^Continuous variables are presented with the interquartile range (IQR).
