# Supplementary material for: Activities of Daily Living Associated with Acquisition of Melioidosis in Northeast Thailand: A Matched Case-Control Study
Source: PLoS Negl Trop Dis. 2013 Feb 21;7(2):e2072. doi: 10.1371/journal.pntd.0002072 (PMC3578767; doi:10.1371/journal.pntd.0002072)
Supplement: Table S3 — Activities associated with melioidosis acquisition by inoculation in the 30 days before onset of symptoms. (DOC) [file pntd.0002072.s003.doc]

**Table S3:** Activities associated with melioidosis acquisition by inoculation in the 30 days before onset of symptoms

| **Factors** | **Cases**  (n=287) | **Controls**  (n=513) | **Conditional OR (95%CI)** | **P value** |
| --- | --- | --- | --- | --- |
| ***Activities related to skin inoculation***  Working in a rice field – % (no) | 72% (208 / 287) | 48% (245 / 513) | 2.9 (2.1-4.0) | < 0.001 |
| Median working duration – hours/week^†^ | 21 (IQR 3 to 56) | 9 (IQR 2 to 49) | 1.1 (1.0-1.1) | 0.02 |
| Median depth of leg submerged in water or  soil – cm ^†^ | 10 (IQR 0 to 30) | 0 (IQR 0 to 20) | 1.2 (1.0-1.3) | 0.01 |
| Trouser length – % (no)^^ |  |  |  |  |
| Above the knee | 23% (48 / 208) | 18% (44 / 245) | 1.0 | 0.01 |
| Knee length | 25% (52 / 208) | 19% (46 / 245) | 0.9 (0.5-1.6) |  |
| Below the knee | 18% (38 / 208) | 16% (39 / 245) | 0.7 (0.3-1.5) |  |
| Ankle length | 34% (70 / 208) | 47% (116 / 245) | 0.4 (0.3-0.8) |  |
| Footwear – % (no) ^^ |  |  |  |  |
| None | 40% (84 / 208) | 24% (60 / 245) | 1.0 | < 0.001 |
| Sandals | 43% (89 / 208) | 44% (107 / 245) | 0.7 (0.4-1.1) |  |
| Boots | 17% (35 / 208) | 32% (78 / 245) | 0.3 (0.2-0.6) |  |
| Gloves – % (no) ^^ |  |  |  |  |
| None | 94% (195 / 208) | 89% (218 / 245) | 1.0 | 0.17 |
| Cloth gloves | 6% (13 / 208) | 11% (27 / 245) | 0.7 (0.4-1.1) |  |
| Washing after working in the rice field – % (no) ^^ | |  |  |  |
| None | 26% (54 / 208) | 18% (45 / 245) | 1.0 | < 0.001 |
| Using water pooled in rice field | 22% (45 / 208) | 13% (31 / 245) | 1.4 (0.7-2.7) |  |
| Using any other water source^‡^ | 52% (109 / 208) | 69% (169 / 245) | 0.5 (0.3-0.9) |  |
| Other activities involving soil exposure – % (no) | 55% (158) | 41% (209) | 1.8 (1.3-2.5) | < 0.001 |
| Other activities involving environmental water exposure – % (no) | 39% (111) | 22% (114) | 2.3 (1.7-3.3) | < 0.001 |
| Walking bare foot – % (no) |  |  |  |  |
| Never | 66% (189) | 74% (380) | 1.0 | 0.01 |
| Less than once a week | 12% (34) | 12% (60) | 1.1 (0.7-1.8) |  |
| More than once a week | 17% (50) | 12% (60) | 1.9 (1.2-2.9) |  |
| Everyday | 5% (14) | 3% (13) | 2.4 (1.1-5.5) |  |
| Water used for bathing – % (no) |  |  |  |  |
| Well water | 8% (23) | 7% (34) | 1.2 (0.7-2.2) | 0.53 |
| Borehole water | 34% (98) | 28% (146) | 1.3 (0.9-1.8) | 0.12 |
| Pond water | 2% (6) | 0% (1) | 11.1 (1.3-92.5) | 0.03 |
| Rain water | 7% (19) | 4% (23) | 1.6 (0.8-2.9) | 0.17 |
| Tap water | 63% (180) | 72% (369) | 0.7 (0.5-0.9) | 0.01 |
| Presence of a wound – % (no) |  |  |  |  |
| No open wound | 77% (222) | 90% (461) | 1.0 | < 0.001 |
| Open wound | 13% (38) | 6% (33) | 2.4 (1.4-4.1) |  |
| Open wound, plus topical application of  herbal remedy or organic substance | 9% (27) | 4% (19) | 2.9 (1.6-5.3) |  |

Estimated odds ratios (OR) are conditional on the matching variables (gender, age, admission date (+/- 2 weeks), and diagnosis of diabetes mellitus). Other activities associated with exposure to soil include non-rice farming and gardening. Other activities associated with exposure to water include fishing and swimming. ^^These risk factors were analyzed as interaction variables to determine whether each factor increased or decreased the risk of working in a rice field. ^†^Continuous variables are presented with the interquartile range (IQR), and the conditional odds ratios shown are for each 10-unit increase. ^‡^Other water sources include wells, boreholes, ponds, rainwater and tap water.
