# Supplementary material for: Activities of Daily Living Associated with Acquisition of Melioidosis in Northeast Thailand: A Matched Case-Control Study
Source: PLoS Negl Trop Dis. 2013 Feb 21;7(2):e2072. doi: 10.1371/journal.pntd.0002072 (PMC3578767; doi:10.1371/journal.pntd.0002072)
Supplement: Table S4 — Activities associated with melioidosis acquisition by ingestion and inhalation in the 30 days before onset of symptoms, and other risk factors. (DOC) [file pntd.0002072.s004.doc]

**Table S4.** Activities associated with melioidosis acquisition by ingestion and inhalation in the 30 days before onset of symptoms, and other risk factors

| **Factors** | **Cases**  (n=287) | **Controls**  (n=513) | **Conditional OR (95%CI)** | **P value** |
| --- | --- | --- | --- | --- |
| ***Activities related to ingestion*** |  |  |  |  |
| Eating food contaminated with soil or dust – % (no) | 40% (114) | 22% (114) | 2.4 (1.7-3.3) | < 0.001 |
| Drinking untreated water – % (no) | 85% (243) | 72% (370) | 2.3 (1.5-3.3) | < 0.001 |
| ***Activities related to Inhalation*** |  |  |  |  |
| Outdoor exposure to dust cloud – % (no) | 62% (179 / 287) | 52% (266 / 512) | 1.6 (1.2-2.2) | 0.002 |
| Median duration of exposure –  hours/week^†^ | 0.3 (IQR 0.1 to 2) | 0.3 (IQR 0.1 to 2) | 1.0 (0.9-1.2) | 0.52 |
| Protection of nose and mouth – % (no)^^ |  |  |  |  |
| None | 82% (146 / 177) | 77% (206 / 266) | 1.0 | 0.50 |
| Hand | 8% (14 / 177) | 8% (22 / 266) | 0.9 (0.4-1.8) |  |
| Cloth | 8% (14 / 177) | 11% (30 / 266) | 0.7 (0.4-1.4) |  |
| Mask | 2% (3 / 177) | 3% (8 / 266) | 0.5 (0.1-1.9) |  |
| Outdoor exposure to rain | 49% (142 / 287) | 30% (154 / 513) | 2.9 (2.0-4.1) | < 0.001 |
| Median duration of exposure –  hours/week^‡†^ | 0.3 (IQR 0.1 to 1) | 0.3 (IQR 0.1 to 1) | 0.9 (0.5-1.9) | 0.88 |
| Protection – % (no)^‡^ |  |  |  |  |
| None | 47% (67 / 142) | 43% (66 / 154) | 1.0 | 0.34 |
| Hat | 41% (58 / 142) | 38% (59 / 154) | 0.9 (0.6-1.6) |  |
| Umbrella | 12% (17 / 142) | 19% (29 / 154) | 0.6 (0.3-1.2) |  |
| History of untreated water inhalation – %  (no) | 23% (65) | 9% (45) | 3.0 (2.0-4.5) | < 0.001 |
| ***Other risk factors*** |  |  |  |  |
| Highest level of education – % (no.) |  |  |  |  |
| Primary school | 85% (243 / 286) | 74% (377 / 512) | 1.0 | 0.001 |
| Secondary school | 12% (34 / 286) | 20% (101 / 512) | 0.5 (0.3-0.8) |  |
| Bachelor degree or higher | 3% (9 / 286) | 6% (34 / 512) | 0.4 (0.2-0.8) |  |
| Monthly income – % (no.) |  |  |  |  |
| < 2,500 baht per month | 43% (123 / 287) | 29% (147 / 513) | 1.0 | < 0.001 |
| 2,500 – 5,000 baht per month | 33% (95 / 287) | 34% (176 / 513) | 0.6 (0.4-0.9) |  |
| 5,000 – 10,000 baht per month | 12% (35 / 287) | 18% (94 / 513) | 0.4 (0.3-0.7) |  |
| > 10,000 baht per month | 12% (34 / 287) | 19% (96 / 513) | 0.4 (0.2-0.7) |  |
| Smoking – % (no) |  |  |  |  |
| Never smoked | 42% (120) | 46% (238) | 1.0 | < 0.001 |
| Former smoker | 22% (64) | 28% (142) | 1.2 (0.7-1.9) |  |
| Current smoker | 36% (103) | 26% (133) | 2.2 (1.4-3.7) |  |
| Alcohol intake – % (no) |  |  |  |  |
| Never | 38% (110) | 36% (186) | 1.0 | 0.13 |
| Former drinker | 31% (90) | 37% (190) | 0.7 (0.5-1.2) |  |
| Current drinker | 30% (87) | 27% (137) | 1.1 (0.7-1.7) |  |
| Any oral steroids – % (no) | 8% (24) | 3% (15) | 3.2 (1.6-6.3) | 0.001 |

Estimated odds ratios (OR) are conditional on the matching variables (gender, age, admission date (+/- 2 weeks), and diagnosis of diabetes mellitus). Untreated water included unboiled water from wells, boreholes, collected rainwater and tap water. ^^These risk factors were analyzed as interaction variables to determine whether each factor increased or decreased the risk associated with exposure to a dust cloud. ^†^Continuous variables are presented with the interquartile range (IQR), and the conditional odds ratios shown are for each 10-hour increase. ^‡^These risk factors were analyzed as interaction variables to determine whether each factor increased or decreased the risk associated with exposure to rain.
