# Supplementary material for: Activities of Daily Living Associated with Acquisition of Melioidosis in Northeast Thailand: A Matched Case-Control Study
Source: PLoS Negl Trop Dis. 2013 Feb 21;7(2):e2072. doi: 10.1371/journal.pntd.0002072 (PMC3578767; doi:10.1371/journal.pntd.0002072)
Supplement: Table S5 — Sources of water consumed and presence of B. pseudomallei in drinking water. (DOC) [file pntd.0002072.s005.doc]

**Table S5.** Sources of water consumed and presence of *B. pseudomallei* in drinking water

| Sources of drinking water | Consumption of water  (information from interview) | |  | Presence of *B. pseudomallei*  (culture positivity per n samples collected) | |
| --- | --- | --- | --- | --- | --- |
|  | Cases  (n=287) | Controls  (n=513) |  | Cases | Controls |
| **Well water** – % (no) |  |  |  |  |  |
| Consumed without treatment | 14% (40) | 9% (44) |  | 11% (1 / 9) | 0% (0 / 18) |
| Consumed after filtration | 0% (1) | 1% (3) |  | None collected | None collected |
| Consumed after boiling | 1% (4) | 1% (7) |  | None collected | None collected |
| **Borehole water** – % (no) |  |  |  |  |  |
| Consumed without treatment | 19% (54) | 12% (59) |  | 13% (4 / 30) | 13% (5 / 38) |
| Consumed after filtration | 2% (6) | 3% (14) |  | 25% (1 / 4) | 0% (0 / 5) |
| Consumed after boiling | 2% (6) | 2% (8) |  | 0% (0 / 3) | 0% (0 / 4) |
| **Collected rainwater** – % (no) |  |  |  |  |  |
| Consumed without treatment | 48% (138) | 34% (176) |  | 0% (0 / 62) | 0% (0 / 71) |
| Consumed after filtration | 4% (12) | 5% (28) |  | 0% (0 / 2) | 0% (0 / 9) |
| Consumed after boiling | 6% (16) | 7% (36) |  | 0% (0 / 4) | 0% (0 / 12) |
| **Tap water** – % (no) |  |  |  |  |  |
| Available but not consumed | 46% (133) | 50% (257) |  | 18% (12 / 68) | 13% (14 / 110) |
| Consumed without treatment | 13% (37) | 10% (53) |  | 13% (3 / 23) | 7% (2 / 30) |
| Consumed after filtration | 5% (14) | 10% (53) |  | 14% (1 / 7) | 0% (0 / 23) |
| Consumed after boiling | 1% (2) | 4% (18) |  | 0% (0 / 2) | 0% (0 / 10) |
| **Bottle water** – % (no) |  |  |  |  |  |
| Consumed without treatment | 14% (40) | 23% (116) |  | 0% (0 / 10) | 0% (0 / 16) |
| Consumed after filtration | 1% (3) | 1% (5) |  | 0% (0 / 2) | 0% (0 / 1) |
| Consumed after boiling | 2% (6) | 2% (12) |  | 0% (0 / 1) | 0% (0 / 2) |
