# Supplementary material for: Activities of Daily Living Associated with Acquisition of Melioidosis in Northeast Thailand: A Matched Case-Control Study
Source: PLoS Negl Trop Dis. 2013 Feb 21;7(2):e2072. doi: 10.1371/journal.pntd.0002072 (PMC3578767; doi:10.1371/journal.pntd.0002072)
Supplement: Text S1 — Supplementary methods. (DOC) [file pntd.0002072.s006.doc]

**Text S1. Supplementary Methods**

**Study design**

Cases were identified from the routine diagnostic microbiology laboratory at Sappasithiprasong Hospital. Patients who were culture positive for *B. pseudomallei* from any clinical specimen during the study period were visited by the study team and screened for inclusion and exclusion criteria. On enrolment of a case, two controls were sought from the hospital computerized record system matched for gender, age (+/- 5 years), presence or absence of diabetes mellitus and admission date (+/- 2 weeks). Individuals who matched on all four criteria were visited by the study team and screened for inclusion and exclusion criteria. Cases and controls were recruited after written and informed consent were obtained.

Each case and control was interviewed to collect information on specified activities of daily living during the 30 days preceding the onset of symptoms relating to melioidosis using a standardized study record form. Relatives were interviewed in the event that the patient was not capable of answering questions. Study staff were trained to administer the questionnaire using both closed and open question techniques. The questionnaire was piloted with professional colleagues and potential cases and controls. These data were not used in the analysis. The questionnaire was edited to minimize the risk of possible misunderstanding or psychological discomfort. Participants were informed that they had right not to answer specific questions, and that they could stop the interview at any point.

The questionnaire contained questions relating to personal information and activities of daily living relating to risk of melioidosis acquisition via inhalation, ingestion and inhalation. Personal information included occupation, home address, family income, and timing of the onset of symptoms. In summary, information on activities relating to skin inoculation included a history of an injury resulting in a wound (defined as at least a break in the skin), recreational activities related to environmental exposure (e.g. gardening), occupation-related exposure (e.g. rice farming, non-rice farming and fishing), duration of exposure to soil and/or water (in hours/week), depth to which the legs were submerged in soil or water (in cm), clothing worn during exposure including any protective gear, washing after exposure including the water source used for this, and the water source used for everyday bathing. Information on activities relating to ingestion included the sources of water consumed (categorized as well, borehole, collected rainwater, tap or bottled water), water treatment prior to consumption (filtration or boiling), and history of consuming food contaminated with soil or dust. Information on activities relating to inhalation included exposure to outdoor dust clouds or being outside in the rain, duration of exposure to dust clouds or rainfall (hours/week), and history of water inhalation (accidental choking/cough fit whilst drinking). Other questions related to smoking, alcohol consumption and steroid intake.

For patients who were seriously ill and patients who died from melioidosis before the diagnosis was made, we interviewed relatives of the participants. If the condition of the participant improved, consent was sought and the interview conducted directly with the participant. Responses were entered into a Microsoft access database with double-data entry.

**Definitions of cases and controls**

Cases were adults aged 18 years or more who were admitted to Sappasithiprasong Hospital and had a new diagnosis of melioidosis based on a culture positive for *B. pseudomallei* from any clinical specimen. Patient with a past history of culture-proven melioidosis were excluded.

Controls were adults age 18 years or more who were admitted to Sappasithiprasong Hospital with non-infectious conditions, and who matched a case for gender, age (+/- 5 years), admission period (+/- 2 weeks), and presence or absence of diabetes. Matched variables are major risk factors for melioidosis, including male gender, increasing age, diabetes and the rainy season. Matching of these known risk factors for melioidosis will improve the probability of defining additional, modifiable risk factors being evaluated in this study. Exclusion criteria of controls were: (1) a previous diagnosis of culture-proven melioidosis, and (2) definite or suspected community-acquired infection associated with systemic inflammatory response syndrome (SIRS). SIRS was defined as 2 or more of the following: (1) Body temperature greater than 38.3**°**C or less than 36**°**C, (2) heart rate greater than 90 beats per minute, (3) respiratory rate greater than 20 breaths per minute, and (4) white blood cell count greater than 12,000/μL, less than 4,000/μL or more than 10% band forms . Patients with SIRS were not eligible as controls, as the sensitivity of culture for the diagnosis melioidosis is not perfect . As a result, culture-negative melioidosis patients were not enrolled as controls*.* Characteristics of cases and controls enrolled into the study were retrieved from the hospital database (Table S2). Cause of illness for controls were described using the international statistical classification of diseases and related health problems, 10^th^ revision (ICD10).

**Sample size calculation**

The formula described by Mark Woodward to calculate a sample size needed to detect an approximate *relative risk* in a case-control study was used . The odds ratio is approximately equal to the relative risk because the prevalence of melioidosis is not high, and this simple sample size calculation for an unmatched case-control design was used because the proportion of discordant pairs is unknown. A two-sided 1% test was used because there is more than one risk factor being tested in the study, and a multivariable conditional logistic regression model was used in the analysis.

We estimated that 65% of the population were exposed to risk factors. We used a ratio of 2:1 in a matched case-control study to identify activities of daily living relating to the risk of melioidosis. To detect an approximate relative risk of 2.0 with 90% power using a two-sided 1% test, at least 231 cases and 462 controls were required. We rounded up the number to 250 cases and 500 controls.

**Statistical analysis**

Continuous variables were presented with the interquartile range (IQR), and the conditional odds ratios were calculated for each 10-unit increase. Working duration, depth of leg submerged in water or soil, trouser length, footwear, gloves and washing after working in the rice field were analyzed as interaction variables to determine whether each factor increased or decreased the risk of working in a rice field. Duration of exposure to dust cloud and protection of nose and mouth were analyzed as interaction variables to determine whether each factor increased or decreased the risk associated with exposure to a dust cloud. Duration of exposure to rain and protection were analyzed as interaction variables to determine whether each factor increased or decreased the risk associated with exposure to rain.

The final multivariable model was developed using a purposeful selection method . Variables associated with acquiring melioidosis at p<0.25 were included as independent variables in a multivariable condition logistic regression model. Variables were removed one at a time from the model if the p-value as determined by the likelihood ratio test was >0.10, least significant variable first, and if they were not confounders. Confounding was determined as a change in odds ratio greater than 20% as compared to that of the full model. To double check that no important factors were removed during the process of deleting and fitting, each deselected variable was tested in turn with the final model and reintroduced into the model if p<0.10.

**References**

1. Levy MM, Dellinger RP, Townsend SR, Linde-Zwirble WT, Marshall JC, et al. (2010) The Surviving Sepsis Campaign: results of an international guideline-based performance improvement program targeting severe sepsis. Intensive Care Med 36: 222-231.

2. Limmathurotsakul D, Jamsen K, Arayawichanont A, Simpson JA, White LJ, et al. (2010) Defining the true sensitivity of culture for the diagnosis of melioidosis using bayesian latent class models. PLoS One 5: e12485.

3. Woodward M (2004) Epidemiology: Study Design and Data Analaysis: Chapman & Hall.

4. Bursac Z, Gauss CH, Williams DK, Hosmer DW (2008) Purposeful selection of variables in logistic regression. Source code for biology and medicine 3: 17.
